# Minor second intervals: A shared signature for infant cries and sadness in music

**DOI:** 10.1177/20416695221092471

**Published:** 2022-04-18

**Authors:** Gabriele Zeloni, Francesco Pavani

**Affiliations:** 83317 Società Psicoanalitica Italiana, Roma, Italy; International Psychoanalytical Association; 9276 Azienda USL Toscana Centro, Firenze, Italy; Center for Mind/Brain Sciences - CIMeC, 19034 University of Trento, Rovereto, Italy

**Keywords:** development, infancy, music, listening, audition

## Abstract

In Western music and in music of other cultures, minor chords, modes and intervals evoke sadness. It has been proposed that this emotional interpretation of melodic intervals (the distance between two pitches, expressed in semitones) is common to music and vocal expressions. Here, we asked expert musicians to transcribe into music scores spontaneous vocalizations of pre-verbal infants to test the hypothesis that melodic intervals that evoke sadness in music (i.e., minor 2nd) are more represented in cry compared to neutral utterances. Results showed that the unison, major 2nd, minor 2nd, major 3rd, minor 3rd, perfect 4th and perfect 5th are all represented in infant vocalizations. However, minor 2nd outnumbered all other intervals in cry vocalizations, but not in neutral babbling. These findings suggest that the association between minor intervals and sadness may develop in humans because a critically relevant social cue (infant cry) contains a statistical regularity: the association between minor 2nd and negative emotional valence.

The link between language, music and emotions has attracted attention of philosophers and scientists for centuries (Arbib, 2013; Juslin & Laukka, 2003; Spencer, 1890). Intensity, tempo and rhythm in music trigger feelings that could reflect typical human behavior in the corresponding emotional states (Bowling et al., 2010). For instance, excitement is associated with energy and speed in both music and everyday behavior. However, the systematic links between emotional states and particular tonal relationships in melodies or chords are much less understood. Classical and folk music in the Western culture predominantly use major 2^nd^ intervals in melodies that evoke positive/excited emotions, but use minor 2^nd^ intervals in melodies that evoked negative/subdued emotions (Bowling et al., 2010). Remarkably, this regularity between melodic intervals (i.e., the distance between two pitches, expressed in musical steps or semitones; see footnote^1^ for a brief primer on the notion of musical intervals) also holds cross-culturally, as evidenced by the comparison of Western music and Carnatic music, the classical music of South India (Bowling et al., 2012). In addition, the emotional expressions conveyed by Western melodies can be recognized above chance level even by listeners from a native African population (Mafa), naive to Western music (Fritz et al., 2009).

What could be the origin of this cross-culturally shared association between emotions and musical intervals? It has been proposed that human vocal expressions and music communicate emotions through a commonly shared code, based on the perceived relation between pitches (e.g., Bowling et al., 2010; Brown, 2000; Curtis & Bharucha, 2010; Juslin & Laukka, 2003; Spencer, 1890). In line with this hypothesis, Bowling and colleagues (2012) asked participants to read monologues or bi-syllabic words using “the quality of their voice to convey either positive/excited or negative/subdued affect” (p.3). For monologue and bi-syllabic recordings alike, 75% of the intervals detected in negative/subdued speech were smaller than a major 2^nd^ (i.e., they were minor 2^nd^ or less), whereas this percentage reduced to less than 50% when studying intervals present in positive/excited speech. Similarly, Curtis and Bharucha (2010) recorded human actresses uttering bi-syllabic speech samples to convey four different emotions (anger, happiness, pleasantness and sadness), and found that participants listening to the intervals extracted from the speech productions associated sadness with minor 3^rd^ and with descending minor 2^nd^.

While these previous works provide initial evidence in support of the hypothesis of a shared code between human vocalizations and music based on musical intervals, it is possible that *intentional* vocal utterances of humans mimicking emotional states are contaminated by cultural influences, especially when linked to *spoken language*. A stronger test of the shared code hypothesis is to assess the proportion of different musical intervals in *non-intentional* (i.e., spontaneous) and *pre-verbal* human utterances. In this work, we analyzed the incidence of musical intervals in infant cry and infant babbling vocalizations – two of the most ontogenetically primitive melodic expressions that humans can produce. Cry utterances are distress social signals, associated with negative emotions more than babbling utterances. The hypothesis of a shared code for conveying sadness predicts a higher proportion of minor 2^nd^ intervals in infant cry vocalizations compared to infant babbling.

## Methods

Infant cry and infant neutral were obtained from two pre-existing and publicly available databases: the Oxford Vocal (OxVoc) Sounds database (Parsons et al., 2014) and the FreeSound database (https://freesound.org). The rationale for choosing audio-traces from these existing databases, rather than performing direct internet searches, was to limit as much as possible any bias in the selection of infant vocalizations.

Vocalizations in the OxVoc databases are audio-traces free from background noise lasting 1.5 s and matched for total root-mean-square (RMS) amplitude for each clip to −25dBFS (decibels full scale). All infants in OxVoc dataset are full-term, healthy, and aged between 6 and 8 months (M = 6.7 months, SD = 0.9; 5 males and 4 females). They were filmed in their own homes during play and feeding sessions with their primary caregiver. Infant cry vocalizations occurred primarily when infants were separated from their caregivers (n = 20, out of 21 in the database; the trace ‘Baby_cry04.wav’ was excluded because it did not contain a discernable melody and was too edgy; mean pitch = 445.54 Hz, SD = 84.81). Infant babbling vocalizations (n = 24, out of 24 in the database) occurred when infants interacted calmly with their caregiver, often during mealtime or play (mean pitch: 347.34 Hz, SD = 122.34).

Cry and babbling vocalizations in the FreeSound database were retrieved using the search function of the website, using the keywords ‘baby cry’ and ‘baby babble or baby babbling’ respectively (results retrieved on: October 3^rd^, 2020). For each keyword search, we included in our dataset the first 5 retrieved audio-files that matched the following pre-determined criteria: (1) duration of at least 30 s; (2) no background noise or sound effects of any sort; (3) clearly identifiable cry vs. neutral emotions; (4) no indications in the filename suggesting an age above 12 months; (5) uploaded by different users (to avoid selecting vocalizations from the same infant). Using these criteria, four files were excluded because vocalizations were not melodic and were too edgy (three for cry vocalizations, one for babbling vocalizations); one was excluded because the vocalizations were not clearly identifiably as babbling and, as a matter of fact, became cry towards the end of the audio-trace; one was excluded because it contained very few sounds in the first 30 s. The resulting dataset included 5 cry vocalizations (mean pitch = 373.04 Hz, SD = 33.75; mean RMS amplitude = −21.50 dB, SD = 2.53) and 5 babbling vocalizations (mean pitch = 314.32 Hz, SD = 23.24; intensity: mean RMS amplitude = −28.34 dB, SD = 4.41), each lasting 30 s. Unfortunately, no information about the infant (age, sex) or the context in which the vocalization was elicited are available in the FreeSound database.

To confirm the emotional content of each vocalization, we examined the perceived dimensions of Distress (resulting from the question “How distress do you think the baby/adult was?”; response provided on a 9-point Likert scale, with −4 corresponding to “not distressed” and + 4 corresponding to “very distressed”) as well as mood attributed to the individual infant (Infant mood; resulting from the question “Please rate the mood of the baby/adult”) or attributed to the listener (Listener mood; resulting from the question “How did you find the sound?”; responses to mood questions were evaluated on a 9-point Likert scale, with −4 corresponding to “very negative” and + 4 corresponding to “very positive”). For the OxVoc database, we used the original ratings obtained by Parsons et al., 2014 (n = 61, 31 females, mean age = 27.6 years, SD = 6.9). For the FreeSound database, we collected all ratings using an on-line survey from performed with Google Form (n = 13, 12 females, mean age = 25.2, SD = 4.2). Each participant listened to the 30 s of each FreeSound audio-trace immediately before providing the ratings.

Melodic intervals in each vocalization were always identified by musically trained human listeners, and transcribed into notes according to the following procedures. For OxVoc vocalizations, each audio-trace was imported at 44.1 kHz sampling rate in the Wave Pad software (version 10.9, NCH Software) and reproduced at normal speed. Based on the heard audio-trace and with the support of a piano keyboard, the notes contained in the vocalization were transcribed into a music score. The transcription focused only on the note pitch, disregarding its duration. Furthermore, the transcriptions indicated the relative positions of notes, not their actual absolute position. In other words, a C3 note could equally represent a C1 or a C5 note. This simplification reflected our interest in the melodic intervals, rather than actual notes. Each vocalization was discussed among the first author (G.Z.) and 2 expert judges (professional musicians and experienced professors at the Florence Music Conservatory “Cherubini”, Florence, Italy), until a collegial unanimous transcription was obtained. The only vocalizations for which such an agreement was not possible was excluded from further analyses (n = 1 neutral vocalization). All judges were naïve as to the purpose of the study. In producing the transcriptions, the judges were allowed to repeat the audio-trace as many times as they wished, and check the heard notes on a piano keyboard. Note that musical experts are trained to distinguish between glissando and notes when listening to music (or vocalization, by extension): thus, when glissando were present only the starting note and the final note were considered in the transcriptions. A visual description of the overall melodic contour of each vocalization produced by an automated procedure for note identification (Manfredi et al., 2018) was also available to the judges if needed. As a matter of fact, this visual description proved entirely superfluous and at times misleading. Hence, all judges performed note identification through audition alone.

The transcription of the FreeSound vocalization dataset (completed as second) followed similar steps, with the following methodological improvements. As first step, the 30 s audio-trace was imported in the Wave Pad software (version 10.9, NCH Software) at 44.1 kHz sampling rate. The sound was reproduced at normal speed, while its entire envelope was visualized. This auditory and visual procedure allowed clear identification of the discreet sections of the audio-file containing sounds, which we will term from now ‘melodic cells’. Melodic cells were all constituted by a group of sounds delimited by clearly identifiably silence pauses. As second step, each melodic cell was played in isolation, repeatedly, until the single perceivable notes were identified. When needed, the melodic cell was reproduced at slowed speed, taking advantage of a software feature that allows to retain the original pitch of the notes (‘check/reproduce at slower speed’ function). Based on the heard audio-trace and with the support of a piano keyboard or guitar, the notes contained in the melodic cell were then transcribed into a music score. As for the OxVoc dataset, the transcription focused only on note pitch disregarding its duration, and the resulting transcriptions indicated the relative positions of notes within each melodic cell, not their actual absolute position. This procedure was again completed by two professional musicians (n = 2) and one of the authors (G.Z.), but using remote file exchange and video-conference due to the COVID-19 pandemic restrictions (October 2020). During the video-conference each professional musician listened to each melodic cell while seeing the proposed transcription. Next, they played the transcription on their own instrument to check whether it corresponded to the audio-trace. Note that the visualization of the melodic contour resulting from the automatic processing software proposed for the OxVoc transcriptions was now removed from the procedure because it was not necessary for expert listeners. Repeated listening of each melodic cell was always allowed. All changes to the original transcription were discussed until a final agreement was reached. No melodic cell was rejected due to lack of agreement.

The transcriptions were converted into melodic intervals, saved into text files and imported into JASP (jasp-stats.org) and R studio (www.rstudio.com) for data analysis and data visualization. The original OxVoc stimuli cannot be re-published, but they are available at the Oxford Vocal (OxVoc) Sound Database upon request (see Parsons et al., 2014). The FreeSound stimuli are available at the following OSF link (osf.io/vfzh9), together with two transcription examples.

## Results

Cry and babbling vocalizations in the two datasets differed in terms of distress, as well as mood attributed to the infant or to the listener. As expected, in the OxVoc and FreeSound databases alike, infant cry vocalizations were associated with greater distress compared to babbling vocalizations. Furthermore, the mood of the infant and the listener were rated as more negative for infant cry compared to infant babble vocalizations (see Table 1, for all comparisons p < 0.0001 on independent sample t-tests).

**Table 1.** Ratings provided for cry and babbling vocalizations in each dataset. All responses were provided on a 9-point Likert scale (Distress: −4 = “not distressed”, + 4 = “very distressed”; Infant mood and Listener mood: −4 = “very negative”, + 4 = “very positive”).

	Cry		Babbling	
	M	(SD)	M	(SD)
OxVoc dataset				
Distress	1,63	(0,66)	−1,80	(0,95)
Infant mood	−2,35	(0,45)	0,37	(0,94)
Listener mood	−1,99	(0,49)	0,36	(0,87)
				
FreeSound dataset				
Distress	2,18	(0,79)	−2,83	(0,95)
Infant mood	−2,28	(0,82)	2,08	(1,12)
Listener mood	−2,28	(0,75)	1,95	(1,07)

Melodic intervals identified in the OxVoc and FreeSound vocalizations were the unison, major 2^nd^, minor 2^nd^, major 3^rd^, minor 3^rd^, perfect 4^th^, perfect 5^th^. These intervals were present in at least 2.5% of all detected intervals. We focused on these intervals for all subsequent analyses. Very few instances of the remaining intervals were identified (i.e., tritone, minor 6^th^, major 6^th^, minor 7^th^, major 7^th^, and octave; less than 2.5% of all detected intervals; see ‘Min’ column in Table 2).

**Table 2.** Percentage of intervals detected in cry and babbling vocalizations in the OxVoc and FreeSound files. Un: Unison; m2: minor second; M2: major second; m3: minor third; M3: major third; p4: perfect four; tt: tritone; p5: perfect fifth; m6: minor sixth; M6: major sixth; m7: minor seventh; M7: major seventh; Oc: Octave.

	OxVoc		FreeSound			
Interval	Cry	Babbling	Cry	Babbling	Min	Max
Un	2,9%	9,8%	9,4%	22,1%	2,9%	22,1%
m2	52,2%	13,7%	39,9%	18,8%	13,7%	52,2%
M2	14,5%	21,6%	22,6%	23,2%	14,5%	23,2%
m3	11,6%	23,5%	14,6%	9,9%	9,9%	23,5%
M3	7,2%	17,6%	5,2%	8,3%	5,2%	17,6%
p4	5,8%	5,9%	3,5%	5,5%	3,5%	5,9%
tt	0,0%	0,0%	0,3%	1,7%	0,0%	1,7%
p5	4,3%	5,9%	1,7%	2,8%	1,7%	5,9%
m6	1,4%	0,0%	1,7%	1,7%	0,0%	1,7%
M6	0,0%	0,0%	0,7%	2,2%	0,0%	2,2%
m7	0,0%	2,0%	0,0%	1,1%	0,0%	2,0%
M7	0,0%	0,0%	0,0%	0,6%	0,0%	0,6%
Oc	0,0%	0,0%	0,3%	2,2%	0,0%	2,2%

**Figure 1.**
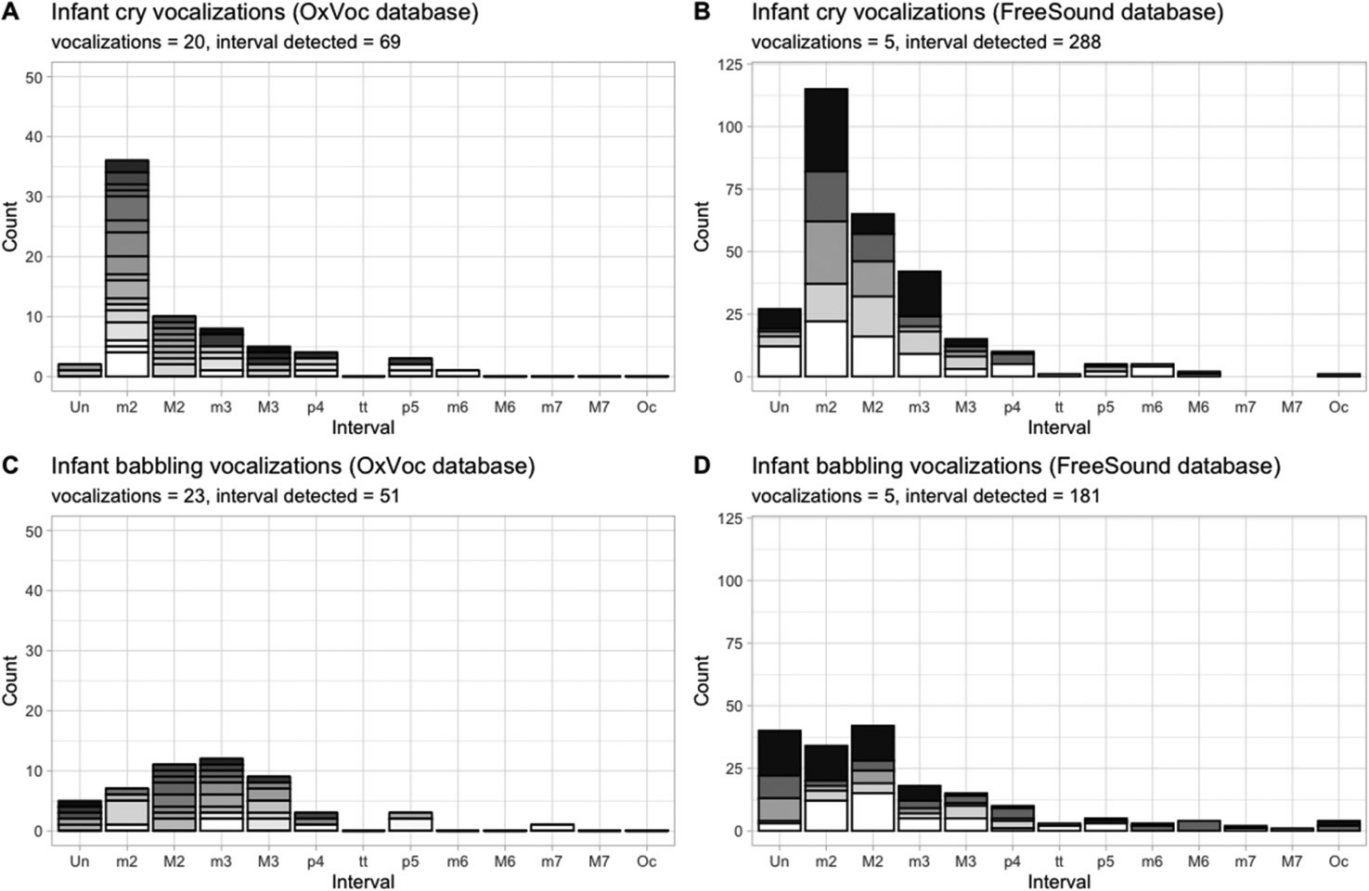
Count of melodic intervals for infant cry and infant babbling, as a function of interval type. (A-C) OxVoc dataset, (B-D) FreeSound dataset. The stacked bars represent count for different audio-files in the dataset. Abbreviations as in Table 1.

Figure 1 shows the count of melodic intervals separately for infant cry and infant babbling vocalizations, as a function of interval type, separately for the two datasets (OxVoc and FreeSound). We first examined whether identified musical intervals were equally represented in cry and babbling vocalizations of the OxVoc dataset (Figure 1, left-side plots). A significant difference in the occurrence of intervals was detected for infant cry vocalizations (Multinomial Test: χ² = 87.85, df = 6, p < 0.001): the count of minor 2^nd^ intervals outnumbered the count of all other intervals in these audio-files (see Supplementary Table S1 for observed and expected counts, with 95% confidence intervals for each interval considered in the analysis; see also Footnote^2^ for limitations of this statistical approach on the OxVoc dataset). No significant difference in the occurrence of intervals was detected for infant babbling vocalizations (χ² = 11.32, df = 6, p = 0.08; see also Supplementary Table S2). In the OxVoc dataset, minor 2^nd^ intervals were present in 17 out 20 audio of infant cry vocalizations (total detected = 36; 52.2% of all measured intervals), whereas they appeared in 4 out of 23 of infant neutral vocalizations (total detected = 7; 13.7% of all measured intervals). The number of ascending and descending 2^nd^ minor intervals (14 vs. 22, respectively) in cry vocalizations was statistically comparable (χ² = 1.78, df = 1, p = 0.18).

This finding was replicated in the FreeSound dataset (see Figure 1, right-side plots). During the 30 s of each vocalization, we identified on average 17 melodic cells (i.e., group of identifiable notes; range 6–35, SD = 9.2), and 46.9 melodic intervals (range 19–73, SD = 18.4). This resulted in a total of 469 melodic intervals overall (288 in cry vocalizations, 181 in babbling vocalizations; t(5) = 2.61, p = 0.059). To study melodic intervals as a function of interval type, we fitted the number of observed intervals in cry and babbling vocalizations separately using a Generalized linear mixed-effect models with Poisson family and log-link function, using Interval Type as fixed effect and Vocalization File as random effect (intercept only). The effect of Interval Type was significant for both cry (Chisq = 138.06, df = 6, Pr(>Chisq) = p < 0.0001) and babbling (Chisq = 43.30, df = 6, Pr(>Chisq) = p < 0.0001) vocalizations, yet the prevalence of minor 2^nd^ interval compared to other intervals substantially differed. In cry vocalizations, the count of minor 2^nd^ intervals outnumbered the count of other intervals (p < 0.001). By contrast, in neutral vocalizations minor 2^nd^ intervals were as represented as major 2^nd^ and unison intervals. The full outputs of these two models are available in Supplementary Tables S3 and S4.

## Discussion

In this work we tested the hypothesis that emotional interpretation of intervals is common to music and vocal expressions. To this aim, we analyzed the incidence of musical intervals between successive recognizable pitches in two types of pre-verbal infant vocalizations: cry and babbling utterances. In two distinct datasets, obtained from on-line repository of infant vocalization traces created independently of the hypothesis of the present study, we found that more than 40% (52% in the OxVoc dataset; 40% in the FreeSound dataset) of the intervals detected in cry vocalizations of pre-verbal infants were minor 2^nd^, whereas this interval was minimally represented in infant babbling vocalizations.

Minor 2^nd^ have often been described as denoting melancholy and anguish (Cooke, 1959; Maher & Berlyne, 1982), in addition they have been associated with negative/subdued emotions in Western music as well as music of other cultures (Bowling et al., 2010; Bowling et al., 2012). This systematic association between the minor 2^nd^ and negative emotions have been interpreted as the consequence of automatic visceral affective response to dissonance (i.e., the way in which sound pairs physically interact in the auditory system), and/or cultural influences. Other works have linked the origin of this association to a common code between music and vocal expression, yet leaving the unresolved the question whether “the voice mimics music, or whether music mimics the voice” (Bowling et al., 2012, p. 7). The present findings show that humans may develop this association because a critically relevant social cue (infant cry) is concurrently characterized by minor 2^nd^ intervals and negative emotional valence. In this respect, they provide support to the common code account and strongly suggest that it is music that mimics the voice.

Why would such regularity occur in infant cry? One possibility is that the aroused state linked to infant cry determines higher pitch vocalizations compared to other utterances, due to the higher tension of the larynx muscles. In turn, this could result in smaller F0 modulations (Wermke et al., 2017) that the human ear categorizes in the smallest musical interval (minor 2^nd^). Under this account, a physical and physiological aspect of infant cry could be at the origin of the prevalence of minor 2^nd^ intervals in its vocalization – with no need to assume any phylogenetic predisposition in determining such a regularity. If these hypotheses were correct, a prevalence of minor 2^nd^ intervals should also be detected in other aroused vocalizations of distress, even when produced by other species such as dogs. Intriguingly, it is already known that negative emotions expressed by yelping dogs are easily recognized by humans (Parsons et al., 2019). Furthermore, one could predict that in those infants in which average F0 values are elevated even further during cry episodes, modulations (hence, intervals) would be even smaller, possibly becoming more difficult to categorize into intervals by the human ear. For instance, this may to the be the case in Autistic Spectrum Disorder (ASD) infants, for whom the average F0 during cry is higher compared to typical infants. These cry vocalizations evoke mental states of uneasiness in caregivers, that may interfere with the adequacy of their parenting response (Esposito & Venuti, 2010).

Future works should extend our preliminary observation in two directions. A first line of development would be to validate our findings using automatic measures of the melodic interval between successive pitches (as in Bowling et al., 2012), rather than using categorical musical-interval identification by expert musicians as here. However, any such automatic method must be able to reliably reproduce the subjective experience of human listeners. In particular, it remains critical to distinguish between the F0 modulations that are actually present in the signal (as detected by an automated proedure) and those that can be perceived by a human listener (musically trained or naïve). The quest for an appropriate automatic processing of infant vocalizations, if successful, could lead to processing large quantity of vocalization data (thus putting our conclusions to a much stronger test), and it would remove any possible bias related to the listeners (thus making the overall investigation more objective). This would allow to thoroughly describe the differences intrinsic to the different vocalizations produced by infants – i.e., intrinsic to the stimulation. A second important line of future developments, however, should examine to what extent these physical changes present in vocalizations are actually parsed and categorized into notes and intervals by listeners. Musical expertise (professional musicians, as here, vs. naïve listeners) or caregiving expertise (e.g., listeners who are parents vs. non-parents) are likely to play a role in these parsing and categorization processes. Moreover, the different degree of musical or parental expertise could also change the degree in which listeners become aware of the specificities of each vocalization and, consequently, associate them with emotional states.

In conclusions, our novel findings support the hypothesis of a commonly shared code for sadness in human vocalizations and music. In addition, while preliminary, they offer a novel interpretation for the association between minor 2^nd^ intervals and the psychological experience of sadness and anguish. Humans are exposed to this association both as adults, while living the experience of assisting infants, but also in their own experience as infants, when the experiencing the high incidence of minor 2^nd^ while crying.

## Supplemental Material

sj-docx-1-ipe-10.1177_20416695221092471 - Supplemental material for Minor second intervals: A shared signature for infant cries and sadness in music Click here for additional data file. Supplemental material, sj-docx-1-ipe-10.1177_20416695221092471 for Minor second intervals: A shared signature for infant cries and sadness in music by Gabriele Zeloni and Francesco Pavani in i-Perception

## Notes

1. Every sound can be characterized in terms of pitch, a perceptual property resulting from sound frequency and associated with the phenomenological experience of the sound being “high” (as notes played at the rightmost end of a piano keyboard) or “low” (as notes played at the leftmost end). In Western music, the distance in pitch between two successive sounds is expressed in conventional steps, known as semitones. On a section of the piano keyboard (see Figure S1 below), examples of semitones are the difference between E (identified with number 5) and F (number 6), or the difference between C (number 1) and C#/Db (number 2). Each interval between two successive notes is identified based on the sum of the semitones between them. In the table of Figure S1, all possible intervals are exemplified with respect to a single note (here the note C, number 1). For the purpose of the present study, it suffices to note that some intervals are termed ‘minor’, others ‘major’, and others are identified with distinct labels. The best way to experience the perceptual consequences of these intervals is to try them yourself, using a free on-line piano keyboard (e.g., https://www.musicca.com/piano). Using the number-to-note mapping provided in Figure S1 compare the effect of playing 1–3 and then 1-2 (a major 2^nd^ and a minor 2^nd^, respectively), or experience the difference between 1–5 and 1–4 (a major 3^rd^ and a minor 3^rd^, respectively).

2. As described in the Methods section, the 20 cry and 23 neutral vocalizations in the OxVoc dataset originated from a total of 9 infants. Hence, some of the vocalizations were recorded from the same infant. Unfortunately, it is not possible to reconstruct which vocalization came from which infant, as this information was not recorded when compiling the dataset (personal communication from Dr. Christine Parson, Nov 2^nd^, 2020). For this reason, we treated the results as cumulative counts and examined them with a multinomial test. This approach is, however, suboptimal because that the multinomial test assumes complete independence between observations. This constitutes a limitation of the analyses on the OxVoc dataset.
