# Supplementary material for: Minor second intervals: A shared signature for infant cries and sadness in music
Source: Iperception. 2022 Apr 18;13(2):20416695221092471. doi: 10.1177/20416695221092471 (PMC9019334; doi:10.1177/20416695221092471)
Supplement: sj-docx-1-ipe-10.1177_20416695221092471 - Supplemental material for Minor second intervals: A shared signature for infant cries and sadness in music [file sj-docx-1-ipe-10.1177_20416695221092471.docx]

Minor second intervals: a shared signature for infant cries and sadness in music

Gabriele Zeloni & Francesco Pavani

**SUPPLEMENTARY TABLES**

Table S1: Observed and expected counts (with 95% confidence intervals for infant cry vocalizations in the OxVoc dataset).

| **Descriptives** | | | | | | | | | |
| --- | --- | --- | --- | --- | --- | --- | --- | --- | --- |
|  | | | | | | **95% Confidence Interval** | | | |
| **Interval** | | **Observed** | | **Expected: Multinomial** | | **Lower** | | **Upper** | |
| Un |  | 2 |  | 10 |  | 0.244 |  | 6.952 |  |
| m2 |  | 36 |  | 10 |  | 27.505 |  | 44.315 |  |
| M2 |  | 10 |  | 10 |  | 4.953 |  | 17.263 |  |
| m3 |  | 8 |  | 10 |  | 3.548 |  | 14.873 |  |
| M3 |  | 5 |  | 10 |  | 1.653 |  | 11.105 |  |
| p4 |  | 4 |  | 10 |  | 1.106 |  | 9.780 |  |
| p5 |  | 3 |  | 10 |  | 0.625 |  | 8.402 |  |
|  | | | | | | | | | |
| Note.  Confidence intervals are based on independent binomial distributions. | | | | | | | | | |

Table S2: Observed and expected counts (with 95% confidence intervals for infant babbling vocalizations in the OxVoc dataset).

| **Descriptives** | | | | | | | | | |
| --- | --- | --- | --- | --- | --- | --- | --- | --- | --- |
|  | | | | | | **95% Confidence Interval** | | | |
| **Interval** | | **Observed** | | **Expected: Multinomial** | | **Lower** | | **Upper** | |
| Un |  | 5 |  | 7 |  | 1.664 |  | 10.907 |  |
| m2 |  | 7 |  | 7 |  | 2.910 |  | 13.370 |  |
| M2 |  | 11 |  | 7 |  | 5.763 |  | 17.981 |  |
| m3 |  | 12 |  | 7 |  | 6.530 |  | 19.085 |  |
| M3 |  | 9 |  | 7 |  | 4.288 |  | 15.718 |  |
| p4 |  | 3 |  | 7 |  | 0.627 |  | 8.274 |  |
| p5 |  | 3 |  | 7 |  | 0.627 |  | 8.274 |  |
|  |  |  |  |  |  |  |  |  |  |
| Note.  Confidence intervals are based on independent binomial distributions. | | | | | | | | | |
|  | | | | | | | | | |

Table S3: Summary of the generalized linear mixed-effect models on number of detected intervals (N_detected) in cry vocalizations using Interval Type (m2, M2, m3, M3, p4, p5, Un) as fixed effect and Vocalization File as random effect (intercept only). In the GLME model the Poisson family and log-link function were selected. Note that minor 2^nd^ intervals are used as baseline in the model, hence all comparisons refer to differences with respect to this melodic interval. All were significant (see list of Pr(>|z|) values in the table).

Generalized linear mixed model fit by maximum likelihood (Laplace Approximation) ['glmerMod']

Family: poisson ( log )

**Formula: N_detected ~ Interval + (1 | File)**

**Data: lm.data.cry**

AIC BIC logLik deviance df.resid

177.2 188.7 -80.6 161.2 23

Scaled residuals:

Min 1Q Median 3Q Max

-2.0707 -0.6325 -0.0793 0.7431 2.7455

Random effects:

Groups Name Variance Std.Dev.

File (Intercept) 0.02033 0.1426

Number of obs: 31, groups: File, 5

Fixed effects:

Estimate Std. Error z value Pr(>|z|)

(Intercept) 3.1254 0.1133 27.577 < 2e-16 ***

IntervalM2 -0.5705 0.1550 -3.681 0.000232 ***

Intervalm3 -1.0073 0.1801 -5.594 2.22e-08 ***

IntervalM3 -2.0369 0.2742 -7.429 1.09e-13 ***

Intervalp4 -1.9681 0.3317 -5.934 2.96e-09 ***

Intervalp5 -2.6800 0.4584 -5.846 5.04e-09 ***

IntervalUn -1.4491 0.2136 -6.785 1.16e-11 ***

---

Signif. codes: 0 ‘***’ 0.001 ‘**’ 0.01 ‘*’ 0.05 ‘.’ 0.1 ‘ ’ 1

Table S4: Summary of the generalized linear mixed-effect models on number of detected intervals (N_detected) in babbling vocalizations using Interval Type (m2, M2, m3, M3, p4, p5, Un) as fixed effect and Vocalization File as random effect (intercept only). In the GLME model the Poisson family and log-link function were selected. Note that minor 2^nd^ intervals are used as baseline in the model, hence all comparisons refer to differences with respect to this melodic interval. The number of detected intervals did not differ between minor 2^nd^, major 2^nd^ and unison interval types (see list of Pr(>|z|) values in the table).

Generalized linear mixed model fit by maximum likelihood (Laplace Approximation) ['glmerMod']

Family: poisson ( log )

**Formula: N_detected ~ Interval + (1 | File)**

**Data: lm.data.babbling**

AIC BIC logLik deviance df.resid

176.9 188.9 -80.4 160.9 25

Scaled residuals:

Min 1Q Median 3Q Max

-2.2324 -0.7764 -0.1130 0.9798 2.1069

Random effects:

Groups Name Variance Std.Dev.

File (Intercept) 0.1413 0.376

Number of obs: 33, groups: File, 5

Fixed effects:

Estimate Std. Error z value Pr(>|z|)

(Intercept) 1.8457 0.2416 7.639 2.18e-14 ***

IntervalM2 0.2113 0.2296 0.921 0.357263

Intervalm3 -0.6360 0.2900 -2.193 0.028335 *

IntervalM3 -0.8183 0.3084 -2.653 0.007975 **

Intervalp4 -1.2238 0.3580 -3.419 0.000629 ***

Intervalp5 -1.5965 0.4794 -3.330 0.000868 ***

IntervalUn 0.1625 0.2321 0.700 0.483773

---

Signif. codes: 0 ‘***’ 0.001 ‘**’ 0.01 ‘*’ 0.05 ‘.’ 0.1 ‘ ’ 1
